# Supplementary figures and images for: Establishment of a quadruplex real-time PCR assay to distinguish the fungal pathogens Diaporthe longicolla, D. caulivora, D. eres, and D. novem on soybean
Source: PLoS One. 2021 Sep 10;16(9):e0257225. doi: 10.1371/journal.pone.0257225 (PMC8432765; doi:10.1371/journal.pone.0257225)

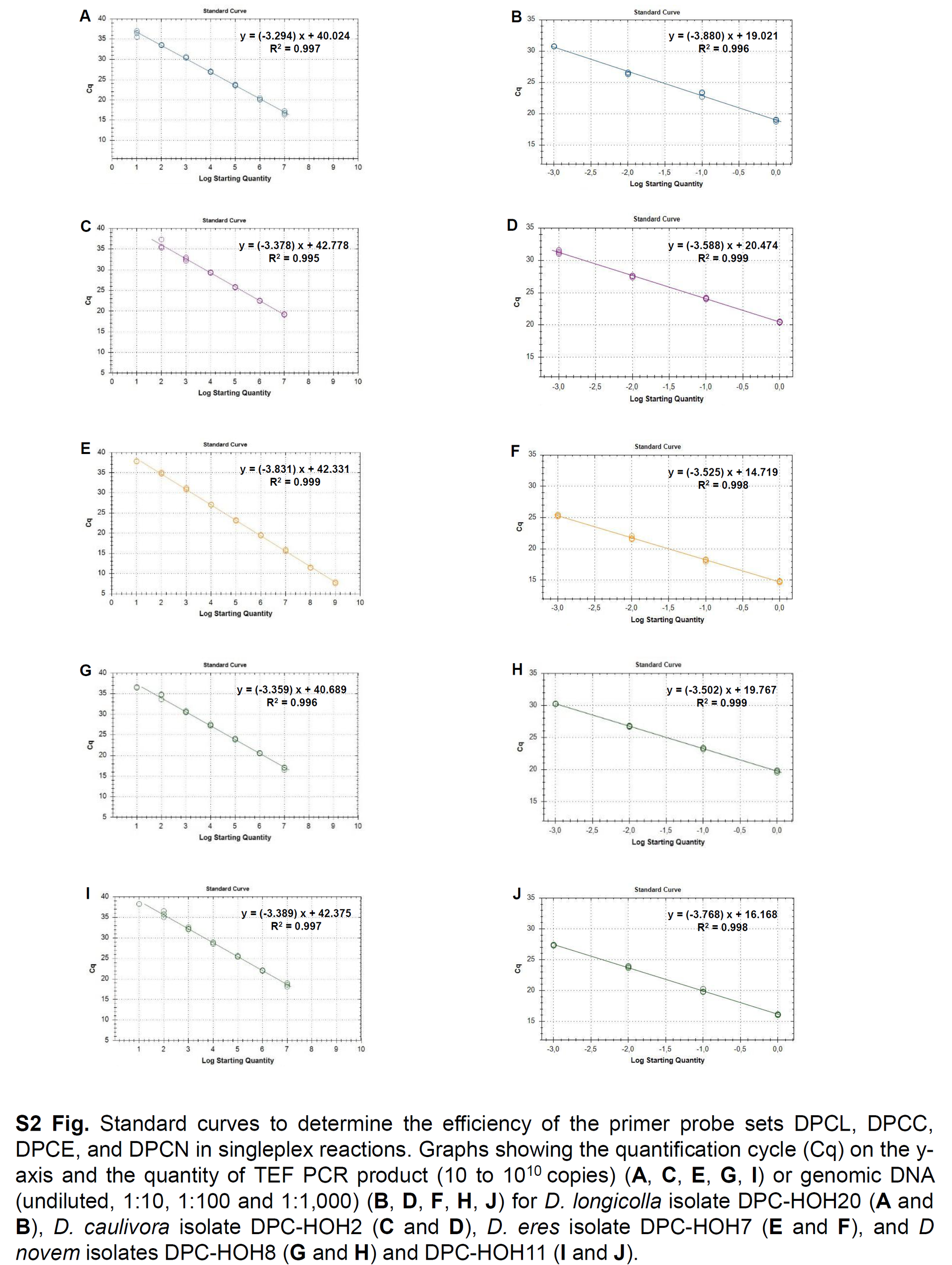

Supplement: S1 Fig — Graphs showing the quantification cycle (Cq) on the y-axis and the quantity of TEF PCR product (10 to 1010 copies) ((A), (C), (E), (G), (I)) or genomic DNA (undiluted, 1:10, 1:100 and 1:1,000) ((B), (D), (F), (H), (J)) for D. longicolla isolate DPC-HOH20 ((A) and (B)), D. caulivora isolate DPC-HOH2 ((C) and (D)), D. eres isolate DPC-HOH7 ((E) and (F)), and D. novem isolates DPC-HOH8 ((G) and (H)) and DPC-HOH11 (I and J). (TIF) [file pone.0257225.s001.tif]

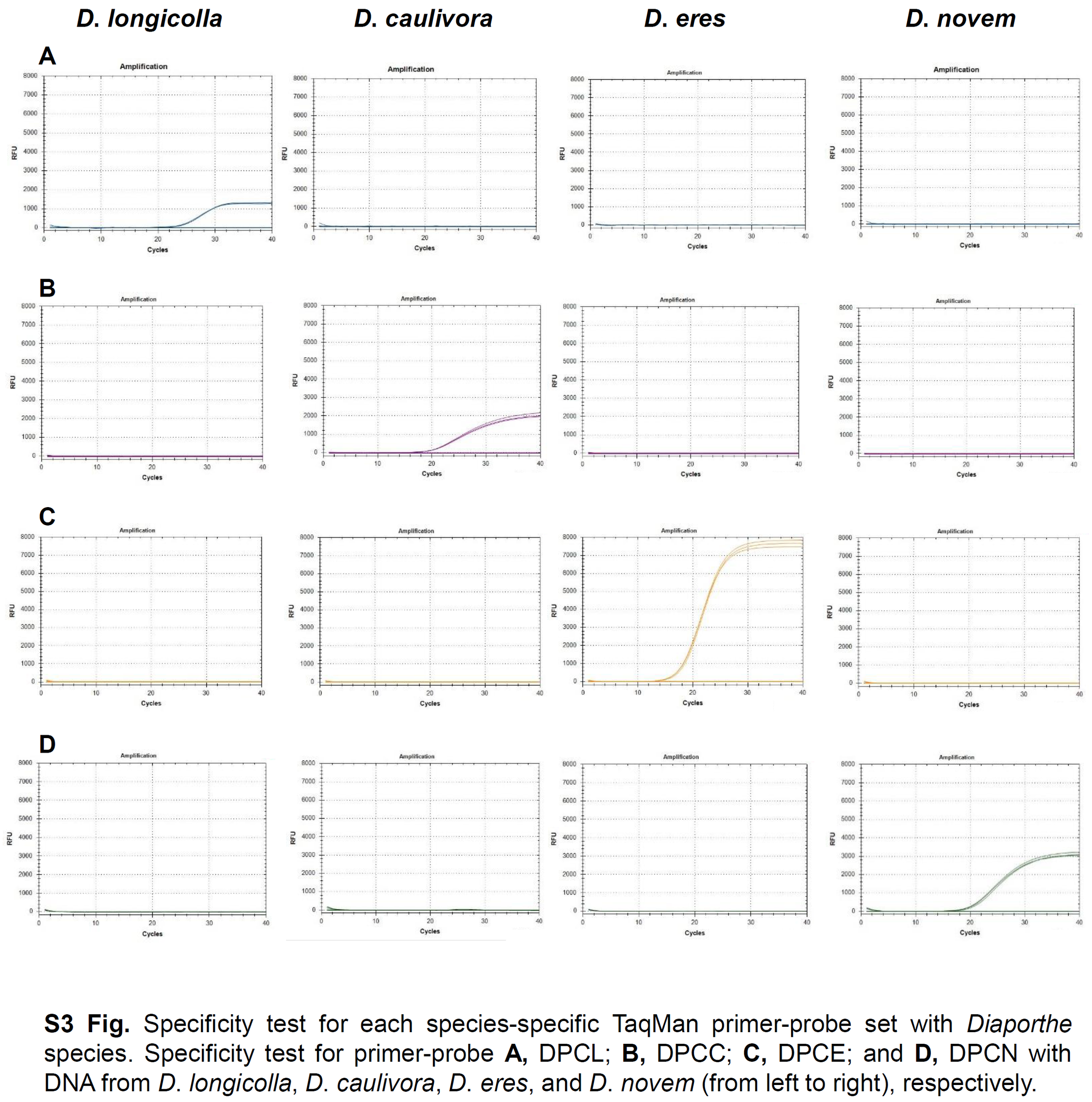

Supplement: S2 Fig — Specificity test for primer-probe set (A) DPCL, (B) DPCC, (C) DPCE, and (D) DPCN with DNA from D. longicolla, D. caulivora, D. eres, and D. novem (from left to right), respectively. (TIF) [file pone.0257225.s002.tif]

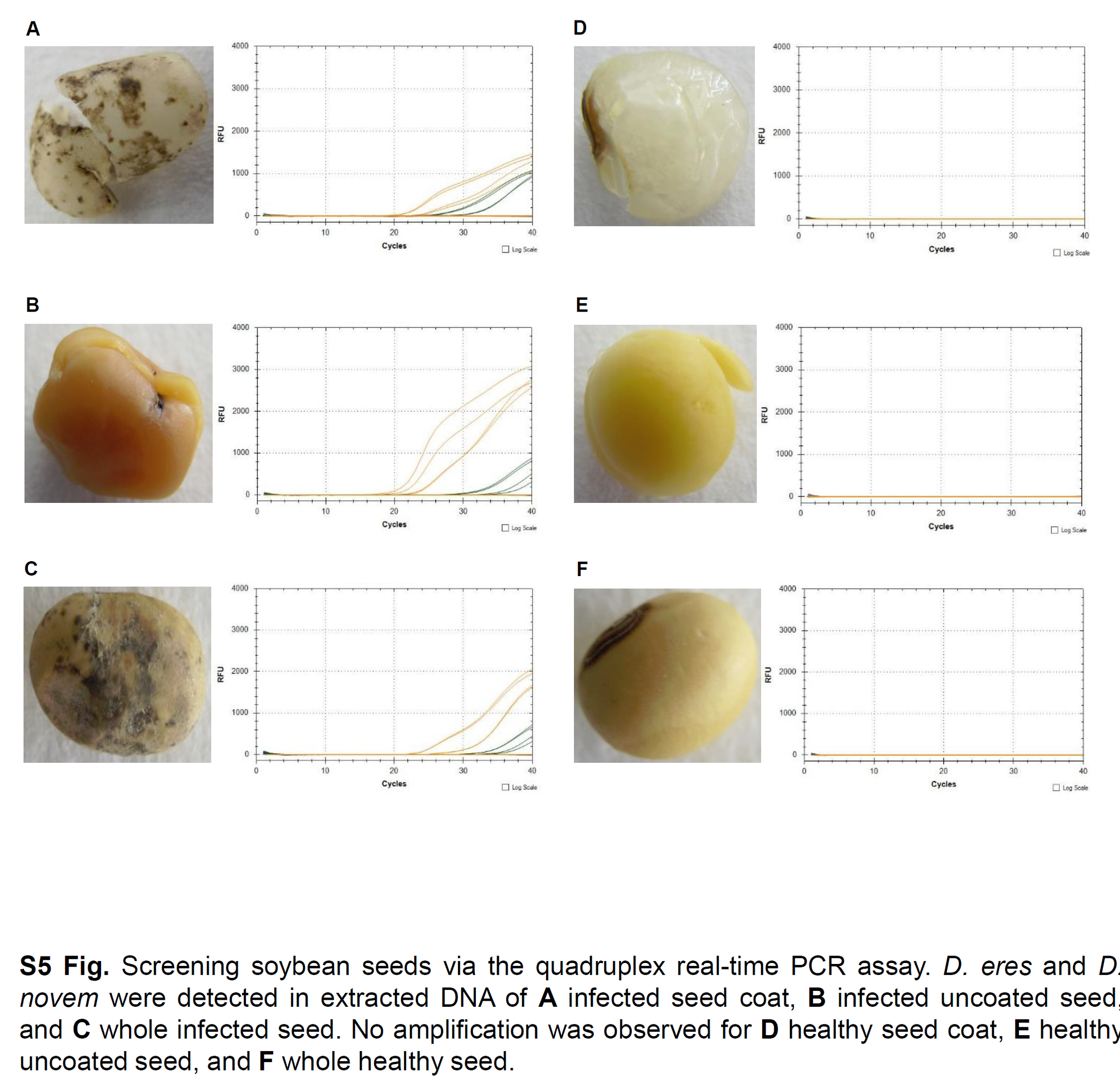

Supplement: S3 Fig — D. eres and D. novem were detected in extracted DNA of (A) infected seed coat, (B) infected uncoated seed, and (C) whole infected seed. No amplification was observed for (D) healthy seed coat, (E) healthy uncoated seed, and (F) whole healthy seed. (TIF) [file pone.0257225.s003.tif]
