# Supplementary material for: Establishment of a quadruplex real-time PCR assay to distinguish the fungal pathogens Diaporthe longicolla, D. caulivora, D. eres, and D. novem on soybean
Source: PLoS One. 2021 Sep 10;16(9):e0257225. doi: 10.1371/journal.pone.0257225 (PMC8432765; doi:10.1371/journal.pone.0257225)
Supplement: S1 Text — (DOCX) [file pone.0257225.s004.docx]

**PrimerBLAST results**

Specificity of the selected primers was tested using NCBI’s Primer-BLAST (<https://www.ncbi.nlm.nih.gov/tools/primer-blast/>). In “Primer Pair Specificity Checking Parameters” we entered nr as database and as organisms *Diaporthe*, *Fungi*, *Pythium*, *Phytopthora*, and *Glycine*. Because the specificity checking only works with primer pairs, not with single oligonucleotides the test was run three times for all primer-probe sets, combining forward primer with reverse primer (FR), reverse primer and probe (RP), and forward primer and probe (FP). The output species were noted for all three combinations; only species returned by both FR and RP were considered to be detected by the set. These species are listed below:

**Primer-probe set DPCL, *Diaporthe longicolla*:**

***Diaporthe longicolla***, *Diaporthe sojae*, *Diaporthe unshiuensis*, *Diaporthe sp*. isolate G.04, *Diaporthe phaseolorum*, *Diaporthe sp.* strain SAUCC194.63, *Diaporthe tectonendophytica*.

**Primer-probe set DPCC, *Diaporthe caulivora*:**

***Diaporthe caulivora***, *Diaporthe phaseolorum* var. *caulivora*

**Primer-probe set DPCE, *Diaporthe eres*:**

***Diaporthe* *eres***, *Diaporthe* *vacuae*, *Diaporthe* *mahothocarpus*, *Diaporthe* *nobilis*, *Diaporthe* *fukushii*, *Diaporthe* *perniciosa*, *Diaporthe* *lonicerae*, *Diaporthe* *castaneae-mollisimae*, *Diaporthe* *biguttusis*, *Diaporthe* *cotoneastri*, *Diaporthe* *phaseolorum*, *Diaporthe* *rosicola*, *Diaporthe* *ellipicola*, *Diaporthe* *neilliae*, *Diaporthe* *alnea*, *Diaporthe* *nitschkei*, *Diaporthe* *bicincta*, *Diaporthe* *celastrina*

**Primer-probe set DPCN, *Diaporthe novem*:**

***Diaporthe novem***, *Diaporthe pseudolongicolla*, *Phomopsis sp.* ER 1657, *Phomopsis sp.* ER 1639, *Phomopsis sp.* JMS-2010g, *Phomopsis sp.* JMS-2010e, *Phomopsis sp.* CBS 117165, *Diaporthe sp.* AG-2020c, *Diaporthe gulyae*, *Diaporthe stewartii*, *Diaporthe cucurbitae*, *Diaporthe subordinaria*, *Diaporthe angelicae*, *Phomopsis sp.* DAR73811, *Diaporthe sp.* YPT-2011a

While the set DPCC seems to be fully specific to *D. caulivora*, all other primer-probe sets also seem to be able to detect other *Diaporthe* species. It needs to be considered, however, that many species names used to be synonymous and the sequences in NCBI might be incorrectly annotated. For example, *D. sojae* and *D. phaseolorum* detected by DPCL are most likely actually *D. longicolla*. The species found by the DPCE primers are even more synonymous. *D. novem* was newly defined, because of this the databases show few synonyms but it cannot be concluded that all the different names found in the primer-BLAST actually represent different species.

Overall the primer-BLAST results are not easily interpreted. It does not seem unlikely that the primer-probe sets can detect other *Diaporthe* species in addition to the ones they were designed for. It is clear, however, that there is no unintentional detection of any species occurring on soybean in Central Europe.
