# Supplementary material for: Establishment of a quadruplex real-time PCR assay to distinguish the fungal pathogens Diaporthe longicolla, D. caulivora, D. eres, and D. novem on soybean
Source: PLoS One. 2021 Sep 10;16(9):e0257225. doi: 10.1371/journal.pone.0257225 (PMC8432765; doi:10.1371/journal.pone.0257225)
Supplement: S2 Text — (DOCX) [file pone.0257225.s005.docx]

**Test of the primer-probe sets in Duplex reactions with both templates**

To ensure adequate efficiencies of the TaqMan primer-probe sets in the presence of other oligonucleotides and fluorogenic dyes, duplex real-time PCR assays were performed. We tested all six combinations of primer-probe sets. Each combination was tested by applying parallel dilution series with DNA of both species. The efficiencies were still acceptable (Table 1). Also, for undiluted (20 ng) and 1:1,000 diluted genomic DNA from both species in individual reactions C_q_ values were recorded (Table 1). To establish how detrimental high concentrations of a different template would be to the reaction we did experiments where low concentrations of one template were combined with high concentrations of the other. Again, C_q_ values were recorded (Table 1). These tests show the ability of the primer-probe sets to discriminate two species of *Diaporthe* in parallel. Applying two different templates does not have a strong impact on the performance of the assay even if the other, competing template was present in much higher concentration than the target template (Table 1).

**Table 1. Duplex real-time PCR assays with primer-probe combinations DPCC/DPCE, DPCN/DPCC, DPCN/DPCE, DPCL/DPCC, DPCL/DPCE and DPCL/DPCN**

| Duplex PCR | Template DNA  Species – Target Isolate | Primer-Probe sets | E(%)^a^ | C_q_ 20 ng DNA^b^ | C_q_ 20 pg DNA^c^ | C_q_ 20 ng and 20 pg DNA^d^ | C_q_ 20 pg and 20 ng DNA^e^ |
| --- | --- | --- | --- | --- | --- | --- | --- |
|  |  |  |  | Same concentration of DNA of both species | | High conc. of DNA of one species combined with a low conc. of DNA of the other species and vice versa | |
| Set 1 | *D*. *caulivora* – DPC_HOH2 | DPCC | 85.7 | 17.9 | 30.0 | 18.2 | 28.7 |
|  | *D*. *eres* – DPC_HOH7 | DPCE | 80.6 | 18.6 | 30.2 | 30.1 | 18.5 |
| Set 2 | *D*. *caulivora* – DPC_HOH2 | DPCC | 85.7 | 17.6 | 29.3 | 17.7 | 28.5 |
|  | *D. novem* – DPC_HOH15 | DPCN | 97.4 | 14.2 | 24.6 | 24.2 | 14.4 |
| Set 3 | *D*. *eres* – DPC_HOH7 | DPCE | 80.6 | 18.1 | 30.0 | 18.2 | 30.0 |
|  | *D. novem* – DPC_HOH15 | DPCN | 97.4 | 13.9 | 24.7 | 23.9 | 14.3 |
| Set 4 | *D*. *longicolla* –DPC_HOH28 | DPCL | 88.4 | 17.8 | 30.4 | 16.7 | 28.9 |
|  | *D*. *caulivora* – DPC_HOH2 | DPCC | 85.7 | 17.7 | 29.5 | 28.6 | 17.9 |
| Set 5 | *D*. *longicolla* – DPC_HOH28 | DPCL | 88.4 | 17.2 | 29.5 | 17.7 | 28.3 |
|  | *D*. *eres* – DPC_HOH7 | DPCE | 80.6 | 18.2 | 29.9 | 29.8 | 18.2 |
| Set 6 | *D*. *longicolla*$-$ DPC_HOH28 | DPCL | 88.4 | 16.0 | 28.2 | 17.5 | 28.8 |
|  | *D*. *novem* – DPC_HOH15 | DPCN | 97.4 | 14.1 | 24.5 | 24.2 | 14.2 |

Each quantification cycle (C_q_) value is the average of technical duplicates.

^a^ Efficiencies of primer-probe sets in the duplex reactions. Dilution series of both species were used; the same dilution for both species.

^b^ C_q_ values measured in reactions where undiluted DNA from both of species was used.

^c^ C_q_ values measured in reactions where 1:1,000 diluted DNA from both of species was used.

^d^ C_q_ values measured in reactions where DNA from the first species in the set was undiluted and DNA from the second species of the set was diluted 1:1,000.

^e^ C_q_ values measured in reactions where DNA from the first species in the set was diluted 1:1,000 and DNA from the second species of the set was undiluted.
