# Supplementary material for: Establishment of a quadruplex real-time PCR assay to distinguish the fungal pathogens Diaporthe longicolla, D. caulivora, D. eres, and D. novem on soybean
Source: PLoS One. 2021 Sep 10;16(9):e0257225. doi: 10.1371/journal.pone.0257225 (PMC8432765; doi:10.1371/journal.pone.0257225)
Supplement: Attachment — Submitted filename: PONE-D-20-38183_reviewer comments.pdf [file pone.0257225.s007.pdf]

Title: Establishment of a quadruplex real-time PCR assay to distinguish the fungal pathogens *Diaporthe longicolla*, *D. caulivora*, *D. eres*, and *D. novem* on soybean

Manuscript: PONE-D-20-38183

Line 34. "…………..the genus Diaporthe (syn. Phomopsis)…………..."

Line 36. Check the authority name for D. longicolla - [Resolving the Diaporthe species occurring on soybean in Croatia (nih.gov)]

Line 89. Check authority names for the fungal names.

Lines 113-115. Why were the other species not tested on the stems? Was there seeds inoculated with these four species?

Line 157. How is the PCR product (efficiency in %) > 100 for DPCE/DE? (Table 2).

Line 172. Define higher efficiencies.

Lines 206. The section on results reads more like a discussion. I would suggest reading research papers related to development of qPCR to rewrite this section. For example, there are no results on the primer blast? Or on the cut-off values for the assays.

Lines 237-238. The authors describe that they obtained good efficiencies and lower efficiencies, it would be better to provide what the Ct values look like. There is no information on the cutoff- values of Ct for each of the primer/ probe combination.

Line 281. Table 4. Please list the Cq cut-off values.

Line 330. More information needed on how much Cq value was obtained on the different species on each of the seed samples tested. How did this compare with the traditional isolation method in terms of fungal recovery?

Lines 384-394. I agree with the thoughts, but the main concern is about Diaporthe eres, which is believed to be a complex of species rather than a phylogenetically distinct species. Any thoughts about this? https://core.ac.uk/display/81525550
